# Selection of Digestive Tract Reconstruction After Partial Gastric Sparing Surgery in Patients With Adenocarcinoma of the Esophagogastric Junction of cT_2_-T_3_ Stage

**DOI:** 10.3389/fsurg.2022.899836

**Published:** 2022-06-30

**Authors:** Junli Zhang, Xijie Zhang, Sen Li, Chenyu Liu, Yanghui Cao, Pengfei Ma, Zhenyu Li, Zhi Li, Yuzhou Zhao

**Affiliations:** The Affiliated Cancer Hospital of Zhengzhou University & Henan Cancer Hospital, Zhengzhou, China

**Keywords:** gastric cance, digestive tract reconstruction, double tract reconstruction (DTR), gastric anastomosis, tnm (8th edition)

## Abstract

**Objective:**

To investigate the appropriate reconstruction method of the digestive tract after partial gastric sparing surgery for adenocarcinoma of the esophagogastric junction of stage cT_2_-T_3_.

**Methods:**

A retrospective analysis of the clinical data of patients with adenocarcinoma of the esophagogastric junction from January 2015 to January 2019 in the General Surgery Department of Zhengzhou University Affiliated Tumor Hospital was performed. Patients with intraoperative double tract anastomosis composed the double tract reconstruction (DTR) group, and patients with intraoperative oesophagogastrostomy with a narrow gastric conduit group composed the oesophagogastrostomy by a narrow gastric conduit (ENGC) group. We analysed and compared the short-term postoperative complications and long-term postoperative nutritional status of the two groups of patients.

**Result:**

There were no statistically significant differences between the two groups of patients in terms of age, sex, preoperative haemoglobin level, albumin level, cT, cN, neoadjuvant therapy or not, pathological type and Siewert type. In terms of BMI and body weight, the ENGC group was higher than the DTR group, but the difference was not statistically significant (*p *= 0.099, *p *= 0.201). There was no significant difference between the two groups of patients in terms of upper resection margin, operation time, blood loss, tumor diameter, pT, pN and postoperative hospital stay. The gastric resection volume of the DTR group was much larger than that of the ENGC group, and there was a significant difference between the two (*p* = 0.000). The length of the lower resection margin of the DTR group was also significantly greater than that of the ENGC group (*p* = 0.000). In terms of surgical approach, the proportion of the DTR group with the abdominal approach was significantly higher than that of the ENGC group, and the difference between the two was statistically significant (*p* = 0.003). The postoperative exhaust time in the ENGC group was significantly shorter than that in the DTR group (*p* = 0.013). However, there was no statistically significant difference between the two groups in terms of anastomotic leakage, anastomotic bleeding, intestinal obstruction, abdominal infection, pneumonia, pancreatic leakage, lymphatic leakage,death within 30 days after surgery, or overall complications. In terms of anastomotic stenosis, the incidence in the ENGC group was higher than in the DTR group, and the difference was statistically significant (*p* = 0.001). There was no significant difference in oral PPI, haemoglobin or albumin levels in patients at 3 months, 6 months, or 12 months after surgery. Comparing reflux/heartburn symptoms at 3 months and 6 months after surgery, we found no statistically significant difference between the two, while in terms of reflux/heartburn symptoms at 12 months after surgery, the findings of the ENGC group were higher than those of the DTR group, and the difference was statistically significant (*p* = 0.045). In terms of poor swallowing, the ENGC group was always higher than the DTR group, and the difference between the two groups was statistically significant (*p* < 0.05). There was no statistically significant difference in body weight between the two groups at 3 months or 6 months after surgery. At 12 months after surgery, the body weight of the patients in ENGC group was significantly higher than that in the DTR group, and the difference between the two groups was statistically significant (*p* = 0.039).

**Conclusions:**

For patients with cT2-T3 stage oesophagogastric junction adenocarcinoma with tumours less than 4 cm in diameter, ENGC anastomosis is recommended for patients with a high tumour upper boundary, with obesity, short mesentery, or disordered vascular arch, and for routine patients, DTR anastomosis is recommended.

## Introduction

In recent years, the statistical results of clinical data from Europe (1), America (1), Japan and South Korea (2), and China (3) all show that the incidence of adenocarcinoma in the esophagogastric junction is increasing annually. In the past, total gastrectomy was usually performed by surgeons for advanced adenocarcinoma of the esophagogastric junction. With the increasing awareness of organ function protection, the majority of surgeons are seeking for the radical surgical treatment of adenocarcinoma of the esophagogastric junction, and at the same time, they are also actively seeking for the appropriate way to reconstruct the digestive tract (4). In terms of the radical treatment of tumors, it has been reported that the lymph node metastasis rates of No.4d, 12a, 5 and 6 patients with T_2_-T_3_ stage upper gastric cancer were 0.99%, 0.006%, 0 and 0 (5), respectively, indicating that the metastasis rates of distal perigastric lymph nodes in such patients were very low, suggesting that patients with T_2−3_ stage upper gastric cancer may not have dissected No. 4d, 12a, 5 and 6 lymph nodes. The results of this study also provide a theoretical basis for proximal gastrectomy in patients with stage T_2−3_ upper gastric cancer. A meta-analysis showed no significant difference of 5-year overall survival rate, recurrence rate between total gastrectomy and proximal gastrectomy for upper-third gastric cancer (6). At present, there are many ways to reconstruct the digestive tract after proximal gastrectomy, and different methods have their own advantages and disadvantages (7). Double tract reconstruction (DTR) can significantly reduce the incidence of reflux oesophagitis (8). However, this procedure involves more anastomotic sites, which theoretically increases the incidence of anastomotic leakage and the cost. Oesophagogastrostomy by a narrow gastric conduit (ENGC) is relatively simple and more suitable for patients with longer oesophagectomy times, but postoperative anastomotic stenosis often occurs. At present, there are few reports comparing DTR and ENGC. Therefore, the General Surgery Department of the Affiliated Cancer Hospital of Zhengzhou University conducted a retrospective study on the above situation to provide a basis for gastrointestinal surgeons to select appropriate digestive tract reconstruction methods for patients with oesophageal and gastric junction adenocarcinoma at stage CT_2−3_.

## Objects and Methods

The general clinical data of patients with adenocarcinoma of the oesophagogastric junction in the general surgery department of the Affiliated Cancer Hospital of Zhengzhou University from January 2015 to January 2019 were retrospectively analysed. All patients underwent surgery by the same group of surgeons. One group was defined as the DTR group, while the other group was defined as the ENGC group. The short-term postoperative complications and long-term postoperative nutritional status were analysed and compared between the two groups. The entry criteria were as follows: (1) preoperative endoscopic pathology confirmed adenocarcinoma of the oesophagogastric junction; (2) the preoperative clinical T stage was cT_2_-T_3_; (3) the maximum diameter of the tumour evaluated by CT at the first diagnosis was ≤4 cm; (4) preoperative examination and intraoperative exploration showed no evidence of distant metastasis, with R0 resection being performed in both cases; and (5) the patients underwent radical proximal gastrectomy. The exclusion criteria included (1) severe patient heart and lung disease that could not tolerate radical surgery and (2) incomplete clinical case data. According to the above entry and discharge criteria, a total of 118 patients with oesophagogastric junction adenocarcinoma were included in this study – 60 patients in the DTR group and 58 patients in the ENGC group. This study was discussed and approved by the ethics committee of the hospital, and all the patients' family members signed informed consent for surgery.

## Methods

### Anastomosis Method

**Figure 1.**
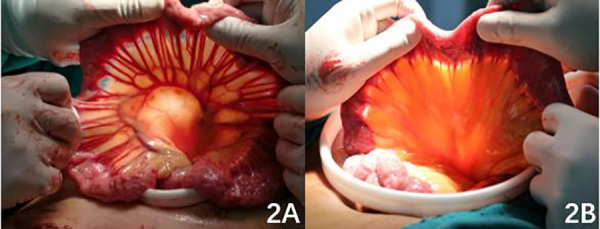
Figure 1 A: postoperative upper gastrointestinal contrast in the DTR group; 1B: postoperative upper gastrointestinal contrast in the ENGC group.

In the DTR group, the jejunum was dissected approximately 15–25 cm from the distal end of the Treitz ligament. The end-to-side anastomosis of the oesophagus and the distal jejunum was completed with a circular stapler with diameter of 23–25 mm before transcolon. The anastomotic site was reinforced with continuous full-thickness barb suture, and the anastomotic site was embedded with a plasmomuscular layer to reduce tension. The jejunum stump was closed and embedded. The stapler base was placed in the jejunum approximately 15 cm from the distal end of the oesophagojejunal anastomosis, and a round stapler with diameter of 23–25 mm was placed through the residual stomach to complete the side-to-side anastomosis of the residual stomach and jejunum. The gastric stump was closed again with a straight-cut closure device, and the anastomotic stoma was reinforced by continuous full-thickness barb suture. Approximately 30 cm from the distal gastrointestinal anastomosis, the anastomosis between the proximal jejunum and distal jejunum was performed with a circular stapler with diameter of 23–25 mm . The anastomosis was reinforced by continuous full-thickness barbed suture, and the mesangial foramen was closed. The postoperative upper gastrointestinal contrast is shown in Figure 1A. In the ENGC group, a tubular stomach with a diameter of approximately 3 cm was made by using a linear cutting closure device, and the residual gastric closure line was embedded with absorbable thread. The bottom stapling seat of the circular stapler was placed at the oesophageal stump, the anterior wall of the appetizer was cut, the circular stapler with diameter of 25–26 mm was placed, and the end-to-end anastomosis of the oesophageal stump and stomach was completed. Absorbable sutures closed the incision of the anterior wall of the residual stomach, and barb sutures continued to strengthen the anastomotic site. The postoperative upper gastrointestinal contrast is shown in Figure 1B.

### Statistical Analysis

SPSS 22.0 software was used for statistical analysis, normally distributed data are represented, and the independent sample t test was used for comparisons between the two groups. The measurement data with a nonnormal distribution are expressed by months (range). Enumeration data are represented by the use case (%), and the *χ*^2^ test was used for comparisons between groups. A nonparametric Z test was used to compare the nonnormally distributed data and grade data between the groups. *p* < 0.05 was considered statistically significant.

## Results

### Comparison of General Information

There were no significant differences between the two groups in age, sex, preoperative haemoglobin level, albumin level, CT, CN, neoadjuvant therapy, pathological type or Siewer classification. In terms of BMI and body weight, the data of patients in the ENGC group was higher than those in the DTR group, but the difference was not statistically significant (*p* = 0.099, *p* = 0.201). The specific results are shown in Table 1.

**Table 1:** General information.

Variables	DTR (*n* = 60)	ENGC (*n* = 58)	*χ*^2^/t/Z	*p*
Age(years)	60.5 ± 9.7	62.5 ± 8.7	−1.250	0.214
Gender			1.250	0.264
Male	47	50		
Female	13	8		
BMI (kg/m^2^)	21.4±2.8	22.2 ± 2.1	−1.663	0.099
Weight (kg)	65.5 ± 7.5	67.5 ± 9.2	−1.285	0.201
Hemoglobin (g/L)	121.9 ± 12.4	122.9 ± 8.2	−0.557	0.579
Serum albumin (g/L)	37.7 ± 3.7	38.2 ± 3.6	−0.771	0.442
cT			0.816	0.366
T_2_	37	31		
T_3_	23	27		
cN			0.136	0.713
N_0_	29	30		
N_+_	31	28		
Neoadjuvant chemotherapy			0.459	0.498
Yes	20	16		
No	40	42		
Differentiation			0.302	0.583
High-middle	28	30		
Low	32	28		
Siewert type			0.833	0.361
II	26	30		
III	34	28		

### Intraoperative and Postoperative Indicators

There were no significant differences between the two groups in terms of upper surgical margin, operative time, blood loss, tumour diameter, PT, PN or postoperative hospitalization time. The volume of gastrectomy in the DTR group was much larger than that in the ENGC group, and there was a significant difference between the two groups (*p* = 0.000). The length of the lower incisions in the DTR group was also significantly greater than that in the ENGC group (*p* = 0.000). In terms of surgical approach, the percentage of the DTR group choosing the abdominal approach was significantly higher than that of the ENGC group, and the difference was statistically significant (*p* = 0.003). The postoperative exhaust time of the ENGC group was significantly shorter than that of the DTR group (*p* = 0.013). The detailed results are shown in Table 2.

**Table 2:** Comparison of intraoperative and postoperative conditions among two groups.

Variables	DTR (*n* = 60)	ENGC (*n* = 58)	*χ*^2^/t/Z	*p*
Volume of the gastric specimen			−8.629	0.000
≥2/3	50	2		
½	10	46		
≤1/3	0	10		
Upper cutting margins (cm)	2.3 ± 1.6	2.6 ± 1.7	−1.056	0.293
Lower cutting margins (cm)	7.1 ± 2.2	5.1 ± 1.6	5.585	0.000
Operative approach			7.284	0.007
Abdominal approach	40	20		
Left combined thoracoabdominal approach	20	29		
Operation time (min)	143.9 ± 20.2	152.1 ± 26.8	−1.869	0.064
Intraoperative blood loss (ml)	128.0 ± 81.7	150.7 ± 68.4	−1.663	0.105
Tumor size (cm)	3.6 ± 1.1	3.8 ± 1.3	−1.102	0.273
pT			−1.146	0.252
T_1_	5	3		
T_2_	31	26		
T_3_	20	24		
T_4_	4	5		
pN			−0.391	0.696
N_0_	31	32		
N_1_	20	18		
N_2_	7	7		
N_3_	2	1		
First anal exhaust time (d)	3.4 ± 1.1	2.8 ± 1.3	2.524	0.013
Postoperative hospital stay (d)	10.7 ± 1.7	10.2 ± 2.7	1.718	0.088

### Results of Complications in the two Groups

There were no significant differences in anastomotic leakage, anastomotic haemorrhage, intestinal obstruction, abdominal infection, pneumonia, pancreatic leakage, lymphatic leakage, death within 30 days after surgery or total complications between the two groups. The incidence of anastomotic stenosis in the ENGC group was higher than that in the DTR group, and the difference was statistically significant (*p* = 0.001). The specific results are shown in Table 3.

**Table 3:** Comparison of postoperative complications among two groups.

Variables	DTR (*n* = 60)	ENGC (*n* = 58)	*χ* ^2^	*p*
Anastomotic leakage	0/60	1/58	1.043	0.492
anastomotic stenosis	1/60	12/58	10.886	0.001
Anastomotic bleeding	0/60	0/58	–	–
Ileus	2/60	0/58	1.967	0.496
Abdominal infection	1/60	1/58	0.001	1.000
Pulmonary infections	6/60	9/58	0.809	0.368
pancreatic leakage	2/60	0/58	1.967	0.496
lymphatic leakage	2/60	1/58	0.308	1.000
Death	0/60	0/58	–	–
Total complications	12/60	19/58	2.478	0.115

### Postoperative Follow-up

There was no significant difference in oral PPI, haemoglobin or albumin levels at 3 months, 6 months or 12 months after the operation. There was no significant difference between the reflux/heartburn symptoms at 3 months and 6 months after surgery, while the reflux/heartburn symptoms at 12 months after surgery were higher in the patients in the ENGC group than in those in the DTR group, with a significant difference between the two (*p* = 0.045). In terms of adverse swallowing, the data of the patients in the ENGC group was always higher than those in the DTR group, and the difference between the two groups was statistically significant (*p* < 0.05). There was no statistically significant difference in the weight of patients in the 3 months and 6 months groups after surgery, while the weight of patients in the ENGC group was significantly higher than that in the DTR group 12 months after surgery, with a statistically significant difference between the two groups (*p *= 0.039). The specific results are shown in Table 4.

**Table 4:** Comparison of postoperative follow-up among two groups.

Variables	DTR (*n* = 60)	ENGC (*n* = 58)	*χ*^2^ /t	*p*
Reflux/heartburn				
3 m after surgery	5/60	8/58	1.749	0.186
6 m after surgery	6/60	13/58	3.364	0.067
12 m after surgery	4/60	11/58	4.020	0.045
Dysphagia				
3 m after surgery	2/60	12/58	8.496	0.004
6 m after surgery	2/60	13/58	9.676	0.002
12 m after surgery	1/60	6/58	3.980	0.046
PPI^a^ therapy				
3 m after surgery	3/60	7/58	1.900	0.168
6 m after surgery	4/60	9/58	2.357	0.125
12 m after surgery	2/60	5/58	1.477	0.224
Weight (kg)				
3 m after surgery	55.7 ± 6.2	58.2 ± 8.7	−1.748	0.083
6 m after surgery	53.5 ± 5.7	55.8 ± 7.9	−1.770	0.079
12 m after surgery	60.7 ± 7.6	65.6 ± 7.3	−2.034	0.039
Hemoglobin (g/L)				
3 m after surgery	108.0 ± 10.5	107.0 ± 7.8	0.585	0.559
6 m after surgery	106.2 ± 10.7	104.4 ± 7.2	1.102	0.273
12 m after surgery	119.1 ± 8.3	120.5 ± 18.9	−0.547	0.585
Serum albumin (g/L)				
3 m after surgery	33.2 ± 3.6	33.6 ± 3.2	−0.619	0.537
6 m after surgery	31.7 ± 4.2	32.3 ± 4.4	−0.753	0.453
12 m after surgery	35.8 ± 3.9	36.5 ± 4.8	−0.784	0.435

a *Proton pump inhibitor.*

## Discussion

To completely remove the lymph nodes that may metastasize and to avoid severe reflux oesophagitis in patients after surgery, in the past, total gastrectomy combined with oesophagojejunostomy was often used by surgeons for advanced cancer of the oesophagogastric junction. However, after total gastrectomy, the digestive and absorption function of patients becomes severely impaired, leading to significant weight loss in patients later (9–11). Therefore, it is an urgent clinical problem for surgeons to preserve part of the gastric tissue and function. A multicentre retrospective study also found that for oesophageal and gastric junction cancer <4 cm in length, the rate of distal perigastric lymph node metastasis was very low, so transabdominal proximal gastrectomy was recommended (12). At present, the methods of gastrointestinal reconstruction after proximal gastrectomy include oesophageal gastric stump anastomosis (13), ENGC, Kamikawa anastomosis, jejunal interposition and DTR. According to the consensus of Chinese experts on the reconstruction of the gastrointestinal tract by proximal gastrectomy (2020) (14), the expert recommendation rate for ENGC was 81.8%, while the expert recommendation rate for DTR was 91.7%. A recent domestic study shows that most surgeons prefer DTR for gastrointestinal reconstruction after proximal gastrectomy (15). In clinical practice, it has been found that for patients with longer oesophageal invasion, DTR oesophagojejunal anastomosis is often limited by the length of the jejunal loop, and high tension anastomosis is likely to occur after anastomosis, which increases the occurrence of anastomotic leakage. Anastomotic stenosis also occurred in patients after ENGC surgery, but a comparative study on the clinical effects of the two anastomotic methods has not been reported.

**Figure 2.**
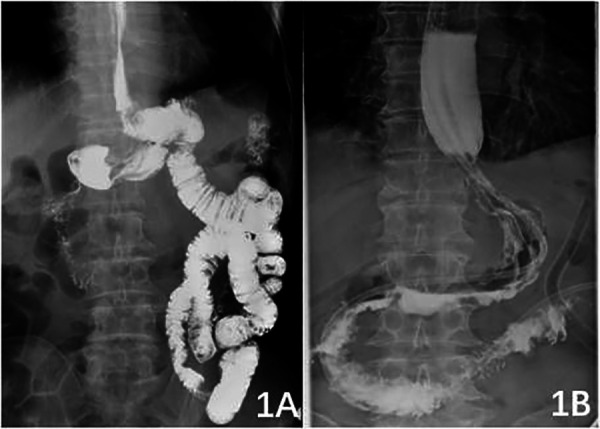
Figure 2A: Relatively long mesentery with regular vascular arches; 2B: relatively short mesentery with disorderly vascular arches.

After retrospective analysis of relevant research results, it was found that the weight and BMI of patients in the ENGC group were higher than those in the DTR group, indicating that the surgeon was more inclined to choose ENGC for obese patients. Patients with normal weight or underweight had longer mesentery and more regular vascular arches (Figure 2A). However, in obese patients, the small mesentery is usually shorter, and the classification of the vascular arch is disorderly (Figure 2B). The anastomotic site tension is heavier after high oesophageal jejunostomy, and ENGC anastomosis is typically selected. Therefore, ENGC is suitable for patients with greater body weight and a higher BMI. Patients in the DTR group had normal or lower body weight and longer mesentery length and did not have these problems. In patients in the DTR group, the volume of gastric excision was larger, while the volume of residual stomach was smaller, thus obtaining a longer lower incision margin. The difference between the two groups was statistically significant. In the patients in the ENGC group, oesophagogastric anastomosis did not have the problem of high tension at the anastomotic site, so it was suitable for patients with a higher upper margin of the tumour, and the upper margin was longer than that of DTR, but there was no significant difference. Patients with Siewert II type had a higher tumour location, so the left thoraco-abdominal combined approach was selected to ensure adequate surgical margins, and high anastomosis was associated with anastomotic tension. The results of this study also showed that the left thoraco-abdominal combined approach was more commonly used in the ENGC group, while the transabdominal approach was more often used in the DTR group, but the difference between the two groups was not statistically significant. With the continuous improvement of surgical techniques and concepts over the years, although more patients in the ENGC group underwent the left thoraco-abdominal combined approach, there was no significant difference between the two groups in terms of operative time and amount of surgical bleeding. The results of this study showed that the exhaust time of patients in the ENGC group was shorter than that in the DTR group, and the difference was statistically significant. It may be that the small intestine was not disconnected in the ENGC group, which ensured the integrity of the small intestinal tract, so the intestinal function recovered faster and the patients' exhaust time was shorter. In terms of postoperative hospital stay, the ENGC group was slightly shorter than the DTR group, but there was no significant difference between the two groups.

In terms of postoperative complications, the incidence of anastomotic stenosis in the ENGC group was higher than that in the DTR group, and the difference between the two groups was statistically significant, which was also consistent with many domestic and foreign literature reports (16, 17). This may be related to the thicker gastric wall. After later endoscopic balloon dilation treatment, the adverse symptoms of swallowing in all patients can be significantly reduced (18). Therefore, our team mainly uses a continuous suture for one round to reinforce the anastomotic site after the completion of the anastomosis of the ENGC group during the operation and does not carry out plasmomuscular layer embedment to reduce the occurrence of anastomotic stenosis as much as possible. For the DTR group, the method of two-layer semianastomosis was continued; that is, after the whole-layer reinforcement of the oesophagojejunal anastomosis, the sarcomuscular layer was embedded in the anastomosis, and stenosis of the oesophagojejunal anastomosis was also rare in clinical practice. This study also found that there were no significant differences in anastomotic leakage, anastomotic haemorrhage, intestinal obstruction, abdominal infection, pneumonia, pancreatic leakage or lymphatic leakage between the two groups. Finally, in terms of total complications, although the data of the patients in the ENGC group were higher than those in the DTR group, there was no significant difference between the two groups, indicating that the operation safety of the two groups was essentially the same except for postoperative anastomotic stenosis.

Previous studies have found that the incidence of reflux/heartburn and adverse swallowing after ENGC is higher than that after DTR (19–21). The same results were also found in the follow-ups of this study. Patients in the ENGC group were worse than those in the DTR group in terms of reflux/heartburn and adverse swallowing, and the difference between the two groups was statistically significant. A study conducted by Japanese scholars (22, 23) found that the incidence of postoperative reflux oesophagitis confirmed by gastroscopy was significantly lower than the incidence of postoperative reflux symptoms. The present study also found that there was no significant difference in oral PPI between the two groups, indicating that in terms of subsequent quality of life, although the incidence of reflux/heartburn symptoms in the ENGC group was higher than that in the DTR group, most patients could tolerate the incidence and did not need PPI drug adjuvant therapy. The weight of patients in the ENGC group was higher than that in the DTR group, and it was found at follow-up that the weight of patients in the two groups still gradually decreased within 6 months after surgery, and there was no significant difference between the two groups. However, at the 12 months follow-up after surgery, the weight of patients in the ENGC group was found to be higher than that in the DTR group. One possible reason is that the majority of patients after gastrointestinal surgery decided to accept subsequent adjuvant chemotherapy, resulting in two groups of patients with 6 months post-operative weight loss. At the end of chemotherapy, the number of symptoms involving digestive tract reactions gradually decreased. In terms of weight gain, the weight difference between the two groups of patients gradually returned to baseline levels. Further study also found that there was no significant difference in haemoglobin or albumin level between the two groups in the postoperative follow-up of 3 months, 6 months and 12 months, which may be because the gastric antrum was retained in both groups, partial gastric function was retained, and the absorption of iron ions was not affected. In summary, the follow-up results indicated that the above anastomosis had the same effect on the recovery of digestive tract absorption function in the later stage, without obvious advantages or disadvantages.

In conclusion, ENGC anastomosis is recommended for patients with cT_2_-T_3_ oesophageal and gastric junction adenocarcinomas with a tumour diameter less than 4 cm and patients with a higher upper boundary of the tumour, obesity, short mesentery of the small intestine, or disorderly grade of the vascular arch. DTR anastomosis is recommended for conventional patients, including those with a transabdominal approach, a low upper boundary of the tumour, and a long mesentery. However, this study is only a single-centre retrospective study with a small sample, and the advantages and disadvantages of the two anastomotic procedures are compared. However, similar data need to be further confirmed in future studies involving larger samples and multiple centres.

## Data Availability

The raw data supporting the conclusions of this article will be made available by the authors, without undue reservation.
